# Teaching EFLLs Listening Subskills With a Speaking-Listening Model in a Computer-Mediated Communication Setting

**DOI:** 10.3389/fpsyg.2022.836013

**Published:** 2022-07-01

**Authors:** Jinman Zhao, Chang In Lee

**Affiliations:** Department of TESOL English Education, Pai Chai University, Daejeon, South Korea

**Keywords:** subskills, micro-skills, macro-skills, speaking-listening model, listening competence, listening strategic competence, CMC

## Abstract

The purpose of this study is to explore the impact of teaching subskills, namely micro- and macro-skills, with a speaking-listening model on the improvement of listening competence. The research included 112 Chinese tertiary students with intermediate English proficiency who were recruited from around the country. Before attending a listening class, the experimental group engaged in oral practice of the subskills, while the control one engaged in conventional listening-oriented preparation before attending a listening class. A randomized controlled trial (RCT), as well as a questionnaire, were used to assess the listening skills. Following the results of the test analysis, we concluded that practicing listening subskills, first verbally and subsequently audibly, had a substantial impact on the development of listening competence. This efficiency was particularly evident when it came to growing discourse and pragmatical listening skills, rather than developing grammatical and sociolinguistic competence. The results of the questionnaire indicated that there was minimal difference between the two groups in terms of listening strategic competence. Our findings were confirmed by coding the interview data, which revealed that tertiary students’ self-agency and class participation had increased. The findings indicate that teaching tertiary students listening with speaking before listening in a computer-mediated communication (CMC) setting has an uneven influence on their development of listening skills.

## Introduction

Listening is a critical component in second language acquisition (SLA). As aural comprehension is fast and not externally observable, listening has been deemed as the most challenging among the four language skills to teach and learn (Vandergrift, 2007; Lynch, 2009; Brown and Brown, 2011; Yang and Chang, 2014). Pedagogical approaches concerning second language (L2) listening acquisition generally have been influenced by two leading hypotheses, Krashen’s input hypothesis (1985, 1992) and Swain’s output hypothesis (1985; 2005). From the input perspective, learners acquire language mainly through comprehensible input, and listening should always be before speaking. From the output perspective, output pushes learners to notice form and is more effective than input in acquiring language form. Therefore, some researchers presume that output should precede input, particularly when learning pronunciation and formulaic knowledge. Experimental studies on the effect of writing-preceding-reading model are rich, but not much on the speaking-preceding-listening. This study aims to investigate the effectiveness of the speaking-preceding-listening model on the acquisition of listening subskills.

As is evidenced experimentally that teaching L2ers subskills for processing input are better than the traditional listen-answer-check approach in preparing learners how to listen (Siegel and Siegel, 2015; Nguyen and Abbott, 2016) and their studies mainly employed the listening-dominated modality. This study was designed to test the speaking-listening model in teaching subskills. Such subskills were also defined as micro- and macro-skills by Brown and Lee (2015, Chapter 15 and 16), and we specified the micro-skills as *prosodic features*, and the macro skills *interaction rules*.

### Target Constructs: Prosody, Interaction Rules in English

Prosody in English is a subset of the sound system that is as complex and unique as any other language in the world. To familiarize L2 learners with the English sound system, both cognitively and physically, our study focused on prosodic features such as sentence stress, intonation, juncture, and linking, as in Brown and Lee’s micro-skills (2015). Respectively, sentence stress gives English its rhythm or “beat” by loudly accentuating some keywords and quietly doing all the others. This is simply because, in English, people do not say each syllable with the same force or strength. They also convey their meaning and attitude with a rising or falling pitch, called Intonation. Equally, they pause their string of speech melodically into thought groups, which is termed Juncture, like a comma in writing. Notably, their pauses are not abrupt, but smooth due to their Linking skill, such as assimilation, reduction, and schwa.

Interaction rules in our study, which mainly consist of negotiation, clarification, attending signals, turn-taking, topic nomination, maintenance, and termination involve the correct use of cohesive devices in expressing intentions. Since such skills are processed at the discourse level to encode meaning. Brown and Lee defined them as macro-skills (2015).

### Rationale for the Two Constructs

First, the two features are shared by both listening and speaking characterizing the sound system. The speaking practice of prosodic features reinforces the listening competence. This point has been verified in SLA research. Chun and his associates stated that the prosodic dimension is a fundamental component of both listening comprehension and oral proficiency (Chun et al., 2008); Haslam posited listening and speaking share the same sound system of spoken language, and learners’ production and perception accuracy should be improved through effective pronunciation instruction (Haslam, 2018). Macro-skills have received less attention in research. However, these skills encourage learners to focus on ideas, make predictions, and listen critically (Wilson, 2018). More research is needed in this area.

Second, Brown and Lee summarized eight oral English characteristics that block listening comprehension (2015, pp. 323–325). As shown in Table 1, clustering, reduced forms, stress, rhythm, and intonation represent prosodic features of English, whereas interaction rules cover almost all characteristics of macro-skills except the advanced macro-skills, like sociolinguistics and strategies. Micro-skills such as phonetic distinction was not included in our research mainly because our samples were at intermediate proficiency, and had finished acquiring the knowledge. Other micro-skills, such as processing speech at different rates of delivery, or with performance variables and redundancy are not taken into consideration because of their non-linguistic-related attributes.

**TABLE 1:** Eight characteristics of spoken language that make listening process uneasy.

Eight characteristics of the English spoken language	
Prosodic features	Clustering
	Reduced forms
	Stress, rhythm, and intonation
Interaction rules	Attending signals and making inference with linguistic and world knowledge, negotiation, clarification, turn-taking, topic nomination, maintenance, and termination
Non-linguistic knowledge	Rate of delivery
	Redundancy
	Performance variables
Advanced linguistic knowledge	Idioms, slang, shared cultural knowledge

Third, to teach English to Chinese EFL learners, the designated features must be instructed in class systematically. EFL learners hardly have chances to possess these subskills within an English environment because they continue to speak their first language (L1) out of class. Chinese and English have standard varieties of prosodic differences (Yu and Gibbon, 2015) as well as the communication styles (Essay, 2018), which pose considerable difficulties for EFL Chinese learners at all levels. The larger the differences are between L1 and L2, the more time is needed for EFL learners to overcome their mother-tongue’s negative interference (Hakuta and McLaughlin, 1996). Therefore, it is pertinent to promote EFL Chinese learners’ familiarity with these English sound features. However, listening and speaking are integrated as one course for many Chinese tertiary EFL learners, and without mandatory oral examinations, oral communication is seldom seen in a listening/speaking class where input practice serves as a paradigm. The result is that a large number of them are weak when it comes to these subskills (Wu, 2019).

## Literature Review

### Listening Is Necessary but Not Sufficient for Aural Comprehension Development

It is well-established that exposure to aural input is necessary for the development of L2 listening skills. Anderson (1995) research on skill learning lends support to the skill acquisition theory in the SLA field. DeKeyser (1998; 2001; 2007) and many others have followed the “skill learning” framework and have advanced their assumptions that language learning, like other skill learning, is first learned as a body of declarative knowledge, then transformed into the procedural knowledge through practice, and automatized through continuous practice. They argue that speaking and listening are asymmetric in terms of procedural knowledge and symmetrical regarding declarative knowledge. The knowledge transferred from one skill can strengthen the storage of the declarative knowledge for developing the other, thus indirectly improving the procedural knowledge of listening. With further practice, it can become an automatized skill. To develop automatized listening skills, declarative knowledge of listening can be stored through listening and speaking, but the listening practice is indispensable for further proceduralization and automization.

In fact, research on input processing suggests that exposure to listening practice alone is not sufficient. This assertion could be illustrated with the following theoretical background. First, in the field of SLA, L2 learners have a limited processing capability (McLaughlin, 2013). It is not possible for them to notice all items during input processing. Second, noticing prerequisites to learning, and more noticing leads to more learning (Schmidt, 1990, 1992, 1994, 2001). During listening, L2 learners are limited in their ability to process spoken input and they only partially attend to the language form for efficient processing of meaning (VanPatten, 1993, 1996; VanPatten and Williams, 2015). More precisely, learners prioritize meaning over processing form during the listening process (Skehan, 1998; Skehan and Foster, 2001). Therefore, limited noticing of form dimensions in input may lead to inadequate learning.

### Integrating Speaking With Listening

#### Theoretical Evidence for the Speaking-Listening Model

Integration of skills is the incorporation of different modality skills that share the same language medium. For example, speaking provides sounds to express messages, whereas listening rests on sounds to understand messages (Hinkel, 2010). It would be more effective for L2 learners to approach English prosodic features and interaction rules with a speaking-listening model than in the listening-oriented one, because listening and speaking are two sides of the same coin and often reinforce each other, as Brown and Lee claims in their seminal work “the integration of at least two or more skills is now the typical approach within a communicative, interactive framework” (2015, p. 316). Similar assumptions can be seen in studies of interaction perspectives (e.g., Pica, 1994; Long, 1996; Gass, 2013). We support this assumption with the following strands of theoretical evidence: (1) the process of articulatory configuration *per se* informs auditory perception. (2) The learner-generated noticing of form in a speaking-listening format is possible. (3) A higher level of cognitive processing (deep processing), such as comparison, clarification, and analysis of language features, may arise when learning activities follow the speaking-listening model.

First, from the perspective of neuropsychology, articulatory configuration informs auditory perception. The sensorimotor (production) system shapes speech perception for L1 acquisition (Sams et al., 2005; Möttönen and Watkins, 2009; Bruderer et al., 2015). Bruderer and his colleagues investigated infants’ speech perception performances when temporarily restraining their articulators with teething toys, and found that those sounds or sound patterns that are in the babbling and productive repertoires could trigger infants to pay particular attention to them during perception. Likewise, the facilitative role of sensorimotor activity in the development of L2 sound perception has been evidenced. For example, researchers of SLA found that the motor areas play an important role in distinguishing phonemes and sentence-level prosodic features in L2 English acquisition (Callan et al., 2004; Gandour et al., 2007; Kato and Tanaka, 2015). Gandour et al. investigated Chinese-English bilinguals learning English prosodic features and reported that the brain domains responsible for phonological processing and speech motor articulation play a crucial role in their performance of chunking auditory sounds at the sentence level (Gandour et al., 2007). Given these studies, it is readily accepted that by producing speech, learners configure specific acoustic signals with specific vocal tracts, and these continuous attempts at adjustment activate the manipulation of the sound systems.

Second, the speaking-listening model is deemed to induce more noticing of language form than the listening-oriented one. This point can be evidenced by two theories: (1) VanPatten’s Primacy meaning principle (1993); (2) Swain’s *noticing function of output* (1985; 1995; 2000; 2005). VanPatten (1990) examined L2 Spanish learners’ ability to process meaning and form in the aural input and concluded that learners in the early and intermediate stages of acquisition tend to adopt a meaning-based approach to input processing (Park, 2013). His Primacy meaning principle reiterates that meaning precedes form in the listening process due to L2ers’ limited attentional resources. In other words, it is difficult for EFL learners to attend to form and meaning simultaneously when listening. This means that during decoding, learners focus on *what is said*, and they extract meaning from form in ways that make sense to them. To this extent, conscious attention to form has adverse impacts on comprehension, particularly for L2 learners at an early stage. Similar findings have been reported by Greenslade et al. (1999); Wong (2001), and Son et al. (2021). By way of contrast, when producing speech, learners’ attention is on *how to say it* since they already know *what to say*. In this encoding process, a message generation starts with a conceptualized preverbal message and then undergoes a formulating process in which the speaker draws on lexical, syntactic, and semantic knowledge to string it together into surface structures (Levelt, 1999). To this extent, encoding prioritizes decoding in terms of noticing form. Considering the *noticing function of output*, Swain expands it to noticing the deficiency of one’s interlanguage (Swain, 1985). Compared with the target language, interlanguage, to some extent, is defective either grammatically or phonologically. To address the imperfection, L2 learners are induced to make cognitive comparisons between their output (the interlanguage) and the relevant input (the target language). This learner-generated noticing of form is a prerequisite for language development. As Schmidt depicted his personal experience in the acquisition of L2 Portuguese, he frequently engaged himself in noticing the *gap* between his output and the input he was exposed to (Schmidt and Frota, 1986), suggesting that the imperfect interlanguage prompts learners to seek out the input with more focused attention and depth of processing (Swain, 1995, 2005; Izumi, 2002).

Third, we assumed that the speaking-listening instructional model would afford L2 learners deep processing of the target language features. Leow (2015, p. 204) defined this deep processing as “the relative amount of cognitive effort, level of analysis, and elaboration of intake, together with the usage of prior knowledge, hypotheses testing, and rule formation, employed in decoding and encoding some grammatical or lexical information in the input.” The speaking-listening model affords a communicative setting, where the interaction *per se*, elicits the depth of processing. According to the interaction hypothesis (Long, 1996), cognitive factors such as *noticing* and *corrective feedback* can be elicited during such interactions as negotiation, clarification, talking about language forms, which are also referred to as LREs^1^. As participants externalize and share their opinions in L2, utterances become objects that can be assessed, agreed, or negated with, and added to or contested in the ongoing flow of activity (Sydorenko et al., 2019). Attempts to make their output comprehensible would force learners to restructure form with more cognitive efforts (Long, 1996). The well-established empirical evidence supports the correlation between higher depth of processing and the amount of L2 development (Leow, 1997; Hulstijn and Laufer, 2001; Rosa and Leow, 2004; Adrada-Rafael, 2017). Therefore, we expected that deep processing may take place when L2 learners are “talking” and “using” the prosodic features, the appropriate interaction rules in our research. As they become familiar with the target language features, their speed of mapping form to meaning during input processing may increase significantly.

#### Empirical Studies for Output-Input Model

Output prompts noticing of input and deep processing of it, leading to effective language acquisition. It was developed from Swain’s (e.g., 1995) *noticing function of output*, which has significantly implicated the pedagogical methods in SLA research. Although research in this area is limited, many empirical investigations do provide positive evidence for the noticing function of output on L2 acquisition. Predominantly, these studies constrain their research within the area of writing to reading model. For example, Izumi (2002) compared the effects of writing and visual input enhancement as attention-drawing devices for adult L2 English learners. The findings showed that the output groups outperformed the input group in the reconstruction and reading tasks. Russell (2014) conceptually replicated Izumi’s experiment by investigating the effect of noticing the function of output for L2 Spanish learners acquiring Spanish future tense. Results revealed that the pushed output followed by exposure to future tense forms in subsequent input enabled learners to learn the target form inductively, whereas the textual enhancement did not, and the output groups outperformed the input group on the comprehension test. The positive empirical evidence on the noticing effect of writing to reading has contributed to recent research on the speaking to listening model.

Due to transient and invisible attributes of speaking and listening, research on the noticing effect of speaking to listening and its learning outcomes is relatively limited. Consequently, little is known about its effect on specific listening competence. About 20 years ago, a negative result was obtained by Izumi and Izumi (2004) when testified the effect of oral modality to listening development. In this case, L2 English learners in the output group were treated to picture description tasks in an input-output model, while learners in the input group were asked to listen and sequence pictures with the corresponding descriptive sentences. Results showed that the input group outperformed the output group on both the production test and interpretation test. However, more positive findings have recently been reported. Linebaugh and Roche (2015) examined the L2 outcomes of speaking to listening after teaching Arabic learners of English three problematic sounds either with an articulatory training or focused aural exposure. By testing learners’ sound discriminating performance, they found that speaking can inform listening to acquire phonetic items. In addition, Zalbidea (2021) investigated the extent to which orally producing L2 impacts learner-generated noticing and grammar development in the subsequent auditory input. By comparing learners’ noticing behaviors in the listening-only group and the speaking-preceding-listening group via stimulated recall protocols, she found that engaging in oral output promoted greater noticing and deeper analysis of auditory input and more robust learning, compared to the no output group.

Empirical research that jointly considers the integrated modalities and single modality on the affordance of noticing and deep processing is limited and not mature yet. Some methodological issues exist in these published resources. First, treatment and testing materials are limited to phonetic items that might be single modality-friendly. It is possible that certain phonetic items might be more speaking-friendly than for listening. Although Zalbidea expands the treatment and testing materials to grammatical items: one future tense, the other clitic. More complicated phonological and oral communicational rules should be employed as treatment materials to support this speaking-preceding-listening model. Second, the extent to which participants engage themselves in the production activities determines the noticing effects of the output and the L2 outcomes. In Izumi’s study, participants of the oral output group were required to do picture description work in the computer lab and a questionnaire within 25 min. It was hard to ensure each participant was fully engaged in the oral output activities due to individual differences such as anxiety or motivation. Moreover, the cognitive processing complexity embedded in the two types of tasks was not equivalent in that the output group just repeated what they heard (mechanically), whereas the input group selected the pictures with the corresponding sentences that they heard (higher cognitive processing). Lastly, previous research constrained their assessment scope within one or two grammatical items, which may not reflect learners’ comprehensive competence in utilizing the target knowledge as a general skill. For instance, Zalbidea adopted sentence acceptability judgment to test L2 learners’ learning outcomes for target grammatical features.

## This Study

Based on the theoretical postulations and previous research findings, we conceived that the effect of the speaking-listening model should be further investigated in an EFL context by systematically treating listening subskills. More importantly, it is necessary to confirm whether all sorts of listening linguistic competence can be developed evenly under this treatment. Furthermore, it is of pedagogical implications to figure out which sort or sorts of linguistic competence grow significantly with this model. Additionally, learners’ perspectives on listening competence development should also be considered in assessing the effectiveness of this model.

This study was devised to address these issues in the EFL Chinese context. Three research questions were developed as the following:

1. What is the impact of the speaking-listening model on listening subskills development in an EFL context?
2. How does this speaking-listening model affect listening competence in terms of linguistic categories?
3. What about the learners’ perception of this speaking-listening model?

To address these research questions with the account of the methodological issues discussed above, Computer-Mediated Communication (CMC), in which teachers and learners conduct instruction and communication in an online environment (Hubbard, 2021), was utilized to improve learners’ speaking engagement. Learners were encouraged to talk and discuss in small virtual groups, which released them from the pressure to speak in front of the whole class. Moreover, the instant feedbacks and practice records bestowed by CMC largely encouraged learners’ engagement. Hubbard pointed out that using CMC activities had a strong effect on stimulating learners’ motivations and seemed to make it easier for shy ones to become involved (Hubbard, 2021). Besides, to foster cognitive comparison and engagement, we used a website application where participants could visualize their productions as sound waves. Second, we expanded the target features from the independent grammatical elements, which were treated and tested in previous studies, to a body of the sound system, and the testing instrument was focal on learners’ comprehensive listening competence. The details are elaborated in the following parts.

## Methodology

### Participants

Intermediate-level EFL learners enrolled in the Listening/Speaking courses at a midwestern university in China. They were randomly assigned to two pedagogical conditions (Speaking-preceding-listening, Listening-oriented) for the RCT. The study is focal on investigating the effectiveness of the oral practice of sound features to the subsequent L2 listening skill development. Some exclusion criteria were applied to ensure that the achievements they made over the semester were maximally due to our designated treatments. Specifically, participants were excluded from the final statistical analysis if their oral class attendance was lower than 70%. Table 2 summarizes language background information in the final sample (*N* = 112; *M_*age*_* = 20.38; 35 female). All participants reported Chinese as their only native language and English as their second language. They had learned English for about 11 (*M* = 11.95) years and generally were at the intermediate proficiency level. The researcher experimented with these samples for two reasons. First, almost all college students in China are now equipped with smartphones or personal computers, which makes it easy for them to access experimental websites and virtual chatrooms to complete speaking and listening exercises. It has been argued that learner participation may be enhanced by using CMC media in both traditional and e-learning settings (Garrison, 2016). Second, these sample students were ready to perform oral and aural tasks because they had some degree of linguistic and cognitive development. Their ability to manipulate abstract linguistic categories and formalize rules and concepts is helpful for language acquisition (Twyford, 1987). Mann–Whitney *U* test of the latest semester’s comprehensive English examination revealed no significant group differences (*p* = 0.06 > 0.05). Further information about the differences in listening comprehension between the two groups is provided in the Testing analysis part. As for the instruction context, the blended model consisting of the online teaching and large classroom teaching with the same local language teacher was adopted in this study. Before the implementation, all the participants signed a consent form suggesting that their participation was voluntary and that the results of the study would not affect their final grades. They were instructed and tested on the following materials.

**TABLE 2:** Participant background information.

	Speaking-preceding-listening	Listening-oriented
	
	M (SD)	M (SD)
N	56	56
Age	20.42	20.34
Years of education in English	11.8	12.1
Latest comprehensive English examination	71.8 (7.48)	69.7 (8.67)

### Materials

#### Instruction Materials

Participants in both the experimental group (EG) and the control group (CG) were instructed with the same textbook: *New Horizon College English---Viewing, Listening and Speaking 3.* Additionally, for the subskills, both groups were introduced to the same online video materials from YouTube^2^. Besides, for homework, all participants were required to find their authentic listening resources based on what they had learned in class. Especially, learners of EG were facilitated with CMC in speaking sessions during the treatment, a speaking practice web^3^ with Automatic speech recognition (ASR), and a voice chat room like *WeChat group (a social software application)*.

#### Testing Materials

Two listening comprehension tests were administrated to both groups at the outset and the end of the study. To ensure the internal consistency between the two tests, a test-retest model or RCT was adopted in that the two-time points for testing were over 2 months. The test material was delivered at normal speed (120--150/min) and consisted of three tasks: (1) the responsive listening task, extracted from *Pearson English International Certificate*^4^; (2) the extensive listening task; (3) the selective listening tasks consisting of two listening Cloze tests, one dialog and one long speech, borrowed from *Tactics for Listening* at Intermediate level (Richards, 2004; see Supplementary Appendix 1).

The test was tallied with integer points; 1–13 questions accounted for two points each, 14∼17 gap-fillings for 3 × 4 = 12 points, and 18∼21 are six points in total. The reliability analysis of a pilot test showed that *Cronbach’s* α = 0.78.

To investigate learners’ language competence underlying their performance, the question items were categorized according to Buck’s framework of describing listening ability (Buck, 2001). As is shown in Table 3, the four listening-competence categories are: (1) *understanding local linguistic meanings*, involving grammatical knowledge such as phonological, lexical, and syntax. This study concerns the basic level of processing speech sound: word recognition; (2) *understanding full linguistic meanings*, involving discourse knowledge, such as cohesion, context, rhetorical schemata, and discourse structure; (3) *understanding inferred meanings*, which involves pragmatic knowledge, such as speech function and illocution force, for interpreting the intended meaning and pragmatic implications; and (4) *communicative listening ability*, which is a higher level of language competence requiring sociolinguistic knowledge of slang, idiomatic expressions, dialects, cultural references, the figure of speech, and registers.

**TABLE 3:** Types of language competence with indicators and the CFA analysis.

Categories	Linguistic categories		Pre-sorted indicators	P (CFA)	Confirmed indicators
(1)	Understanding local linguistic meanings	*Grammatical knowledge:* *phonology, stress, intonation, vocabulary, and syntax*	Q6, Q14, Q15, Q16, Q17	0.817 <0.001 <0.001 <0.001 <0.001	Q14, Q15, Q16, Q17
(2)	Understanding full linguistic meanings	*Grammatical knowledge;* *discourse knowledge*	Q1, Q4, Q11, Q18, Q19, Q20, Q21	0.420 0.565 0.001 0.002 0.098 <0.001 <0.001	Q11, Q18, Q19, Q20, Q21
(3)	Understanding inferred meanings	*Grammatical knowledge;* *discourse knowledge; pragmatic knowledge*	Q2, Q3, Q5, Q8, Q9, Q10, Q13	<0.001 0.008 0.211 0.906 0.004 <0.001 <0.001	Q2, Q3, Q9, Q10, Q13
(4)	Communicative listening ability	*Grammatical knowledge;* *discourse knowledge; pragmatic knowledge; sociolinguistic knowledge*	Q1, Q2, Q4, Q7, Q8, Q10, Q12	0.294 0.231 0.172 0.058 0.056 0.788 <0.001	Q7, Q8, Q12

Each category composed of multiple items is expected to measure the same type of linguistic competence. To demonstrate the reliability of this categorization, the pre-selected indicators were analyzed using confirmatory factor analysis.

The report of CFA in Table 3 shows that there were outliers with *p*-values much greater than 0.05. These outliers were discarded to ensure that all the indicator variables were statistically related to their respective factors. For instance, in the factor group of grammatical knowledge, indicator variable Q6 was removed due to *p* = 0.82 > 0.05, while the other four question items were significantly associated (*p* < 0.05). Similarly, *Q1* (*p* = 0.42), *Q4* (*p* = 0.57), and *Q19* (*p* = 0.1) in factor 2; *Q5* (*p* = 0.21), *Q8* (*p* = 0.91) in factor 3; and *Q5* (*p* = 0.77), *Q10* (*p* = 0.79), *Q2* (*p* = 0.23), *Q4* (*p* = 0.17), *Q1* (*p* = 0.29) in factor 4, were dropped. The confirmed question items were: *Q14, Q15, Q16, Q17*; *Q11, Q18, Q20, Q21*; *Q2, Q3, Q9, Q10, Q13*; and *Q7, Q8, Q12*.

#### Questionnaire

Immediately after the post-listening test, a questionnaire was conducted on two groups to mirror learners’ competence in using listening strategies and their self-efficacy regarding their listening competence. It was developed from Brown and Lee’s list of micro- and macro-skills of listening comprehension (2015) and some popular questionnaires on listening strategies (Vandergrift et al., 2006; Vandergrift and Tafaghodtari, 2010; Bonyadi et al., 2012; Kassem, 2015) consisting of 38 statements both in English and Chinese (see Supplementary Appendix 2). By using the Likert scale, learners’ ideology of using listening strategies was gauged regarding six subsets following Oxford’s (1990) strategy system. According to Oxford (2003), memory-related strategies for listening comprehension pertain to shallow processing level, which relies on techniques to combine sounds and representations. Cognitive strategies rest on reasoning, analysis, summarizing, synthesizing, and formally practicing structures and sounds. Compensation strategies rely mainly on indirect signals to make up for missing knowledge to regulate learning activities according to one’s learning style preferences, monitor, evaluate the task, and success pertaining to meta-cognitive strategies. Socio-affective strategies help learners identify mood and anxiety levels and make adjustments. According to Bernhardt (1997), self-efficacy concerns the belief in one’s competence that determines the goal setting and efforts or willingness. The scales reckoned the perceived agreement with statements along six-point integer scales ranging from 1 (strongly disagree) to 6 (strongly agree), with no neutral position in between to avoid hedge. Of the current 38 statements in this study, 27 items were borrowed from Kassem (2015) with the Cronbach’s α = 0.947. The rest 11 items from Brown and Lee (2015), Cronbach’s α = 0.969. Thus, the 38-item questionnaire was of high internal consistency.

As presented in Table 4, the reliability of the six subsets in their respective classifications was estimated by Cronbach’s α values. Question items embedded in the six subsets were assessed with high internal consistency (Cronbach’s α_1_ = 0.81; α_2=_0.95; α_3=_0.80; α_4=_0.91; α_5=_0.83, α_6_ = 0.85). The relatively high internal consistency of the six subsets ensured that the high reliability of the questionnaire was measured with a higher reliability.

**TABLE 4:** Classification of strategies.

Strategies	Statement items	Cronbach’s α
Memory	13, 15, 29, 32	0.805
Cognitive	1, 2, 7,10, 26, 27, 28, 31, 33, 34, 35, 37, 38	0.953
Compensation	3, 8, 23, 36	0.798
Meta-cognitive	4, 5,11,17, 20, 21, 22, 24	0.909
Socio-affective	6, 9, 25	0.834
Self-efficacy	12, 14, 16, 18, 19, 30	0.851

#### Interviews

Along with the close-ended questionnaire, some face-to-face informal interviews were conducted in a free-talk mode. The interviews were mainly about learners’ perceptions of the relationship between speaking subskills practice and their listening improvement, which were carried out by the teacher at the end of the semester after the class for the experimental group only. In total, 15 students participated in the interviews. The objective interviewees were selected according to their demographic and psychographic features; 4 female and 11 male students from different virtual chatrooms were interviewed. According to the teachers’ feedback, many of them were active in class, and two were relatively introverted.

### Procedure

The classes (both EG and CG) were held once a week for 2 h each in classrooms equipped with computer technology for CMC. EG had access to the designated website (see text footnote 3) and voice chat room like *WeChat*, while CG employed CMC through watching and listening. The randomized controlled trial lasted for 12 weeks.

**FIGURE 1:**
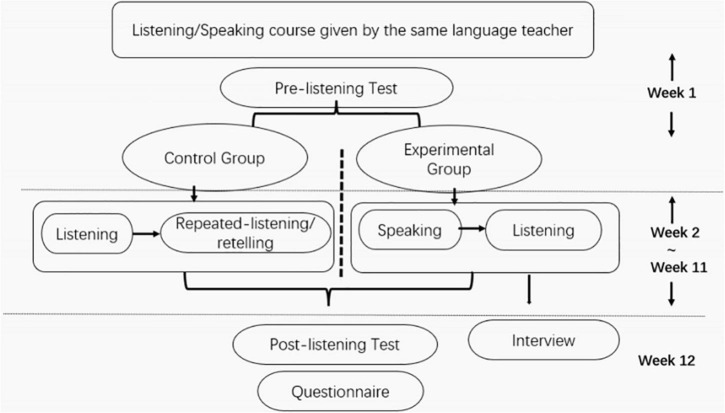
Data collection schedule.

It can be seen that the RCT was conducted and completed following the schedule in Figure 1. In week 1, the teacher introduced the teaching syllabus and research aim and asked students to sign a consent form. Besides, both groups took a mock test before they completed the pretest. From Week 2 to Week 11, EG received instructions with the speaking-listening model while CG with the conventional listening-oriented instruction model. In week 12, both groups took a post-listening test and a questionnaire. The interviews were conducted only with the experimental group.

**FIGURE 2:**
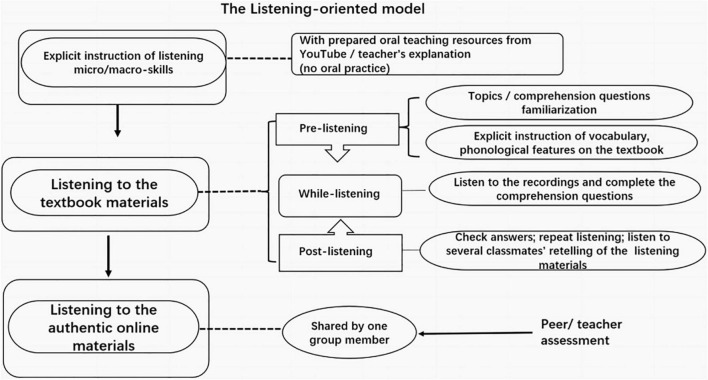
Illustrations for the listening-oriented model.

Learners of the CG were instructed with the listening-oriented method illustrated in Figure 2. As for the knowledge of listening subskills, they received the explicit instruction of online resources on the computer, but without oral practice. For the textbook, they followed the conventional model: pre-listening, while-listening, and post-listening activities. Particularly, the teacher introduced the topic and explicitly taught new vocabularies, some phonological knowledge in the textbook at a pre-listening stage, and learners read the prepared comprehension questions in the textbook. Following which was the listening stage, without or with little teacher’s intervention. Repeated listening was conducted if needed. For the post-listening activities, learners did the comprehension questions and checked with their teacher. Apart from two or three learners’ retelling of the listening materials of the textbook, there was no other oral practice over the class time. In general, all the was much like a *listening-checking-listening* procedure. As assignments, learners were required to find authentic online resources for group listening.

**FIGURE 3:**
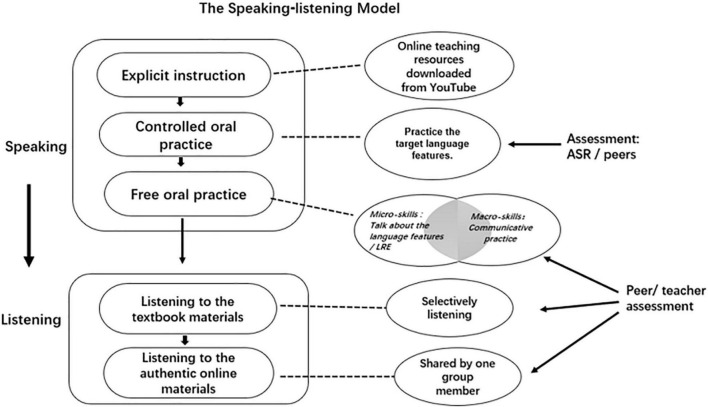
Illustrations for the speaking-listening model.

In the speaking-listening format with CMC displayed in Figure 3, participants received explicit instruction from the prepared online teaching resources at first. Different from the control group, they conducted shadowing exercises with CMC as a controlled oral practice of the subskills. The practice of the target subskills can be repeated on the website where they can compare their production with the given one either aurally or visually. It was followed by a free oral practice session, where group members completed the speaking tasks in a virtual room. The task-based talks, such as pronunciation tactics, and assessing their peers’ work concerning the language features can be operated within CMC. Learners were required to upload their satisfactory free talks into the *WeChat* virtual group, where all their submissions over the semester were stored and assessed by the teacher and peers. Since learners’ engagement in the group work could be reflected in the virtual group, the teacher, as well as group members themselves, could trace their performances and attendance.

The overall syllabus for both CG and EG is shown in Table 5. A total of seven units of the textbook plus micro- and macro-skills were covered over the semester. Noting that the seven units were instructed to the EG in the listening session following the similar pre-while-post-listening model as with CG. The textbook material in each unit was selectively covered for EG since the speaking session took half of the class time.

**TABLE 5:** The overall teaching syllabus for both groups.

Period	Teaching syllabus for CG & EG
Week 1	Introduction of the research goal and so some mock Test; Pre-listening comprehension test
Week 2	Micro-skills: stress + listening to unit 1 of the text book
Week 3	Micro-skills: intonation + listening to unit 2
Week 4	Micro-skills: juncture/linking + listening to unit 3
Week 5	Micro-skills: chunks and formulaic sequence + listening to unit 4
Week 6	Review Micro-skills + listening to unit 4
Week 7	Macro-skills: cohesive devices 1: narration + listening to unit 5
Week 8	Macro-skills: cohesive devices 2: discussion + listening to unit 5
Week 9	Macro-skills: communicative functions 1: negotiation, clarification + listening to unit 6
Week 10	Macro-skills: communicative functions 2: turn-taking, topic nomination/maintenance/termination + listening to unit 6
Week 11	Review of macro-skills + listening to unit 7
Week 12	Post-listening Test & Questionnaire/Interview (EG only)

**FIGURE 4:**
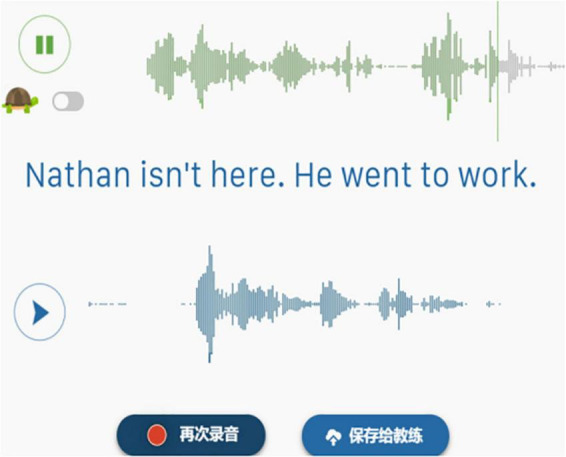
Controlled oral practice with CMC.

To be specific, the speaking session for EG was conducted in the following steps:

1. Watch and learn a prosodic feature, *Linking*, with a prepared video clip^5^;
2. Practice *Linking* with sentences on the given website, and make comparisons between IL and TL. For example, after the target sentence “*Nathan isn’t here. He went to work.”* was played aurally and visually, the learners were required to shadow the sentence, and their oral productions would be recorded and transferred into sound waves for comparison (see Figure 4).

3. Talk about the tactics or learning anecdotes about *Linking* within virtual groups; peer assesses group members’ assignments. For example, learners assessed their peer members’ work and talked about how to put this sentence smoothly “*Some of the students can say it loudly.”* If needed, the teacher joined in their group discussion.
4. Find related online resources and upload them into the virtual chatroom for listening in step 5.
5. Listen to recordings of unit 3 in the textbook as well as the shared audio resources shared by the group members.

### Data Analysis Instruments

To testify the comparability between the two groups’ listening competence, an independent *t*-test analysis was adopted twice, one for the pretest and the other for the post-test. The aim of analyzing the pretest scores was to investigate whether the discrepancy in the two groups’ listening proficiency before treatment was significant or not. The analysis of the post-test scores was to assess the effects of two treatments. In terms of the specific achievements that each group made, a paired *t*-test analysis was conducted within groups. This analysis aimed to identify whether the improvements of one group were significantly larger than that of the other group.

As for the 38-item questionnaire, the classified items were analyzed by independent *t*-test analysis, with the purpose to compare two groups’ listening strategic competence concerning six factors. The reports of face-to-face informal interviews were coded with the English versions.

## Results

### Treatment Effects Assessed by Comprehensive Listening Competence

To address Research Question 1 regarding the effectiveness of two types of treatment, the pre- and post-listening test results were compared and analyzed between the control and experimental groups.

As is shown in Table 6, the mean scores and standard deviation were computed to establish the source of the difference between groups. Specifically, on the pre-listening test, the two groups’ performances were of minimal difference in terms of the mean scores (CG, *M* = 21.1; EG, *M* = 21.7) and standard deviation (CG, Sd = 9.41, EG, Sd = 9.15), indicating that their listening competence was at the similar level, and their later performance was comparable. After the instruction intervention over a semester, the mean score differences on the post-test between the two groups were larger (CG, *M* = 22.9; EG, *M* = 26.2). Regarding the standard deviation, CG (*Sd* = 7.31) was a little lower than EG (*Sd* = 7.52). Further analysis was conducted to test whether the results were statistical or not by independent *t*-test.

**TABLE 6:** Descriptive statistics of the pre- and post-tests.

Groups	Listening comprehension test					
	Pre-test			Post-test		
	*N*	Mean	Std	*N*	Mean	Std
Control	56	21.1	9.41	56	22.9	7.31
Experimental	56	21.7	9.15	56	26.2	7.52

Table 7 presents no significant difference between the CG and EG in the pre-listening test, *t* (110) = 0.34, *p* = 0.74 > 0.05, confirming that the listening proficiency was not statistically different between the two groups before treatments. This means the two groups were comparable in our study. On the post-listening test, a statistically significant difference was found *t* (110) = 2.35, *p* = 0.02 < 0.05, confirming that the discrepancy in listening proficiency was large between the two groups after they received different instruction treatments over the semester. It can be concluded that the teaching effects were different. Therefore, we conducted the paired *t*-test to further analyze how effective the two treatments were.

**TABLE 7:** A comparison of score discrepancy between two groups by *t*-test analysis.

		Statistic	*df*	*p*
Pre-test	Student’ s t	0.336	110	0.738
Post-test	Student’ s t	2.345	110	0.021

As is presented in Table 8, the results of the CG are *t* (55) = −1.75, *p* = 0.09 > 0.05, indicating that the achievements that learners made in this group over the semester were not statistically significant. Cohen’s *d* (effect size) = −0.23, implied that this conventional listening instruction for the control group took effect, but was not meaningful. On the other hand, the results from the EG suggest that learners had made statistically significant achievements at the level of *p* < 0.001. Cohen’s *d* = −0.79 indicated that the instruction on EG had a larger effect on the learners’ performance in the test. Additionally, to investigate the power, we conducted a *post hoc* analysis, and the Power (1-ß err prob) = 0.98 > 0.80, confirming that the sample size is sufficient to support the novel format’s effect.

**TABLE 8:** A comparison of group achievements using Paired *t-*test analysis.

Groups	Tests	Statistic		*df*	*p*	Effect size		Power
Control	Pre/Post	Student’s t	−1.75	55.0	0.086	*Cohen’s d*	−0.234	
Experimental	Pre/Post	Student’s t	−5.92	55.0	<0.001	*Cohen’s d*	−0.791	0.98

### Treatment Effects Assessed by Specific Listening Competence

In response to Research Question 2, we calculated and compared the two groups’ achievements about the four categories of language competence.

Table 9 shows that learners of CG and EG made similar proportions of achievements (about 23%) on language competence category 1, *understanding the local linguistic meaning*, but when it comes to the other three types, their achievements were different. In category 2, *understanding the full linguistic meaning*, learners of the EG made an extraordinarily high improvement with 59%, compared with CG with 18.7%. In terms of the more advanced competence in categories 3 and 4, the differences between the two groups are moderate. Respectively, EG (13.8%) performed better than CG (6.3%) in category 3 in using pragmatic knowledge to infer the underlying meaning, while CG (18.4%) slightly outperformed EG (14.1%) regarding the sociolinguistic skill.

**TABLE 9:** Analysis of achievements within the four categories.

Language competence		1. Understand local linguistic meaning	2. Understand full linguistic meaning	3. Understand Inferred/pragmatic meaning	4. Understand socio-interactive meaning
Components of question items		14, 15, 16, 17	4, 11, 18, 20, 21	2, 3, 9, 10, 13	7, 8, 12
Improvements	CG	22.70%	18.70%	6.28%	18.42%
	EG	23.16%	58.80%	13.82%	14.12%

**FIGURE 5:**
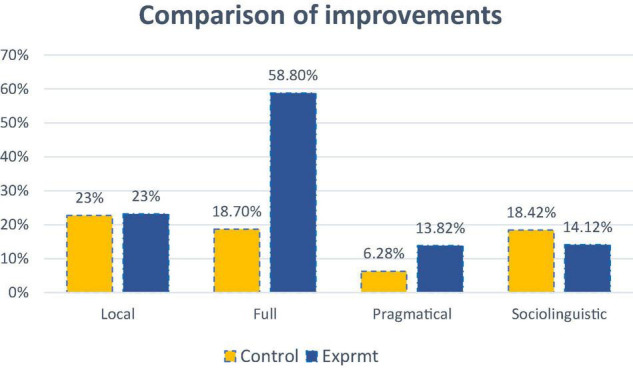
A comparison of improvements regarding the four categories of language competence.

On *understanding full linguistic meaning*, EG made an obvious improvement, about three times higher than CG in Figure 5, indicating EG’s discourse skills, such as understanding cohesion, context, schemata, and discourse structure, grew significantly in this novel format. For a category to *understand the pragmatical linguistic meaning*, EG outperformed CG two-folds. It indicates that this novel format was effective in preparing learners to infer situations, participants, and speaking functions also of the construct: *interaction rules*. However, regarding *understanding local linguistic meaning*, there was minimal difference between the two groups, suggesting that the two kinds of teaching models might take a similar effect in facilitating L2 learners to acquire such knowledge as phonology, spoken vocabulary, oral syntax, etc. Concerning the more advanced *sociolinguistic* knowledge, namely cultural references, figure of speech, and idiomatic expressions, CG outperformed EG, though not obviously. It indicates that the new model was not as effective as the traditional model in assisting learners to acquire advanced cultural-related sociolinguistic skill.

### Treatment Effects Assessed From Learners’ Perceptions

To answer Research Question 3, we conducted a post-only Likert scale *questionnaire* and some face-to-face *interviews* to mirror learners’ self-reflection of their listening strategies, self-efficacy, and class engagement at the end of the research. All participants (112 in total) from two groups were required to submit their answers to the questionnaire, while interviews were only from the experimental group.

#### Questionnaire

**FIGURE 6:**
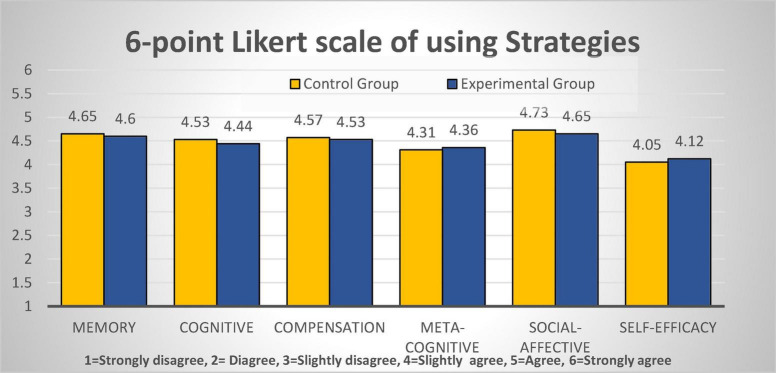
Comparison of means of reported listening strategies from the two groups.

As is shown in Figure 6, learners of both groups positively reported that they employed the listening strategies in processing listening. Yet, there was no clear difference between the two groups in their reflection on using listening strategies. On the self-efficacy, both groups reported comparatively lower scores (<4.3), though EG reported slightly higher CG by about 0.1 point. The independent *t*-test analysis indicates that there was no statistically significant difference among all the five strategies and the self-efficacy at the level of *p* > 0.05, confirming that there was no statistical difference between the two groups regarding learners’ self-reflected listening strategies competence and self-efficacy levels.

#### Interviews

To further decipher learners’ perceptions of the novel model several face-to-face informal interviews were adopted and analyzed for EG. The interviews centered on the content of the speaking practice, and its role in developing listening skills as well as learning motivation. Learners expressed that (1) they scarcely noticed the spoken features before receiving the course; (2) after practicing these features orally at first, they found they could recognize more frequently used sound features, and they were adapting themselves to melodic spoken English; (3) they were more active in completing the self-listening assignments; (4) they were encouraged to assess the group members’ work, and more open to others’ assessment; and (5) they expressed that they benefited from the systematic instruction of all subskills. The following excerpts were from five interviewees:

After the speaking practice (language features), I got to know that not English is not said word by word. It seems ……many words stick together as a character in Chinese. When listen, I think, I just need to get the key word. I would not feel nervous any longer when I miss one or two words. Even though I may miss some, I can still make up by predicting. (Excerpt 1)

I remember that, I easily became distracted whenever listening to, you know, a longer speech. Because I feel/felt defeated by the strange, how to put it? …speech stream. Yes. It’s hard to follow it. But, after I am a little bit familiar with its pace now, I found… that listening to English is like to listening to songs. (Excerpt 2)

I like surfing the internet for my favorite English resources. If my group members like them too, I would become happy. In other words, it is a kind of, uh, achievement, no, it’s an honor for me that my work to be selected listening is talked and valued by my classmates……. (Excerpt 3)

I didn’t wanna say something, something about others’ oral work, because I worried about…. to hurt others directly or indirectly. However, after I found … well, as long as my word was nothing but good for improving language ability, others, I mean, it is welcome by others. Now, my group members are frankly discussing others’ work, and we progress together……. (Excerpt 4)

Actually, the online videos are good, and it, they are helpful, interesting. From watching them, I got to know…uh, each unit is important. I tell myself, don’t skip any unit, any class. I learned much from watching and imitating the native speakers. And now I still keep …keep on watching English movies. For some good sentences, I would imitate again and again. But, you know, it’s not enough. I will talk more in and out class in English. (Excerpt 5)

## Discussion

Regarding research question 1, the results showed that EG outperformed CG on the post-test of the listening comprehension task, suggesting the effectiveness of the speaking-listening model in developing L2 learners’ listening competence. Based on the theoretical hypotheses on the role of verbal production (Krashen, 1985, 1992; Swain, 1995, 2000, 2005; Long, 1996; Leow and Mercer, 2015), we can predict that practicing prosodic and interaction features first orally, and subsequently audibly could prompt greater noticing and deeper processing than the traditional model. The findings are not in line with Izumi and Izumi (2004) findings that the output group failed to outperform the input group by examining learners’ grammatical knowledge: relative clause. Instead, the current findings align with previous research on the positive role of the output-input model on L2 outcomes (Izumi, 2002; Russell, 2014; Linebaugh and Roche, 2015; Zalbidea, 2021). Our findings extend previous research on the role of the speaking-listening model in facilitating L2 learners’ acquisition of language knowledge, from certain phonetic features to systematic sound systems, and from some grammatical patterns to frequently used oral interaction rules. The potential noticing and deep processing function of verbal production have been further confirmed in helping tertiary learners acquire listening subskills in the EFL Chinese context (effect size = 0.79). More specifically, the findings suggest that the beneficial functions of verbal production can be extended to facilitate L2 learners *noticing* and *deep processing* sound features, giving way to robust L2 outcomes that are observable in comprehensive auditory measures. This research lends support to the critical role of oral output in listening development in SLA, which is well recognized theoretically but has not been experimentally evident to date.

Research question 2 asked how the speaking-listening model affected the development of listening competence in specific linguistic categories: grammatical skill, discourse skill, pragmatical skill, and sociolinguistic skill. The results of the study revealed that the novel treatment had an uneven impact on developing listening skills at four different levels. Specifically, results from the first category indicated that both types of treatments were similarly effective in developing L2 learners’ word recognition, a fundamental phonological skill. These findings are inconsistent with the findings of Linebaugh and Roche (2015). The researchers attributed the result to two factors: the demographic characteristics of the subjects and the methodological imperfections. According to the critical period hypothesis (CPH) of SLA, up until the onset of puberty, L2 learners are likely to acquire language skills comparable to those of native speakers (Lenneberg, 1967). It is possible that L2 tertiary learners past puberty are no longer to acquire subtle phonological features at the word level through oral modality. Considering that CPH has been a controversial issue, as some experiments have found negative results (see Long, 2006), we prefer to attribute these similarities to our methodological problem. In this study, the subskill of segmenting word units using phonological knowledge was addressed in the first session of the study, and the test was administrated approximately 5 weeks later, thus the effect might be moderated by the delayed testing factor. The results from the *discourse* and *pragmatic* categories suggest that this novel format had a strikingly positive effect in developing L2 learners’ listening competence, compared to the listening-oriented model (3:1; 2:1), which are in line with the theoretical hypotheses and previous research in this area (e.g., Zalbidea, 2021). The findings could be explained by Swain’s noticing function of output (1995) and Leow and Mercer’s depth of processing (2015), whereby oral output drives L2 learners to attend to the ways of stringing sound units when keeping an effective communication. The fundamental oral communicational rules, such as cohesion, context, speech function, and the like, could be efficiently noticed and processed with depth through oral communication activities, which in turn, promotes the mapping speed of form and meaning during audial input. In sum, the target teaching materials, *prosody*, and *interaction rules*, could be more effectively acquired through the speaking-listening model than the listening-oriented one in terms of improving L2 learners’ discourse and pragmatic listening skills. The findings confirm the previous hypotheses that integrating speaking with listening within a communicative, interactive framework can efficiently reinforce each other (Brown and Lee, 2015). Concerning the last category, *sociolinguistic skill*, the findings indicate that the new format for sociolinguistic skill development was not so effective as the listening-oriented one (7: 9). This, once again, appears consistent with the notion that noticing the function of output is positive for L2 outcomes. Participants in the experimental group were seldom exposed to the culture-related sociolinguistic output, thus they were relatively less attentive to the relevant features in the post-test. These findings lend support to the *Noticing hypothesis* that more noticing leads to more learning (Schmidt, 1994). Furthermore, sociolinguistic skill is multifaceted and would be hard to approach without integration with L2 culture. Scholars suggest that culturally relevant books, and listening/video materials are ways for L2 learners to acquire sociolinguistic features if face-to-face or online communication with native speakers is not possible (Mede and Dikilitaş, 2015). In this study, oral interactions took place between non-native speakers and the cultural elements of English might not have been exploited to a great extent. In this vein, the findings suggest that to acquire culturally relevant sociolinguistic skills, the input may be more effective if output activities are restricted among non-native speakers.

The results of research question 3 regarding the learner feedback on their listening comprehension indicated that both groups responded similarly on listening strategies and did not statistically differ in their levels of self-efficacy, but interviews with the experimental group revealed increased self-efficacy and classroom engagement. As has been postulated that, to prepare learners with strategic competence, teachers should model strategic thinking and students should practice the strategies in new tasks. The findings inform that, without explicit demonstration of strategies use, L2 learners still fail to use listening strategies consciously even after acquiring automatic, proceduralized listening subskills. However, the results are plausible with Buck’s claim that differences in individual performance on listening tests are generally due to differences in linguistic competence rather than differences in strategic competence (Buck, 2001). As for the self-efficacy and classroom engagement, reports from the interviews suggested that the new format was positive in the target context. As is known, most Chinese universities face challenges in encouraging classroom engagement because tertiary L2 learners no longer participate as actively in classroom discussion as they did before puberty. With CMC, the speaking-listening model appears to be operative in breaking the ice and building collaboration, especially with introverted students. It implies that learners’ self-agency to manage their learning can be stimulated in the speaking-listening format.

## Implications and Conclusion

Before presenting the broader implications of this research, some limitations should be acknowledged. First, given the gap between L1 and L2, this study selectively chose four sound features of *prosody* and four *interaction rules*, which are crucial for L1 Chinese learners of English. The target instructional features should be carefully considered given the issue of validity and reliability due to language differences. Second, the target sample was Chinese tertiary EFL learners with easy access to CMC and online resources, and the extent to which the observed results are representative of different age groups of EFL learners is uncertain. Therefore, further research is needed to strengthen our understanding of the impact of the novel format on the development of learners’ listening competence. Additionally, the grammatical skills of the experimental group in word recognition still need further investigation due to possible memory decay in the post-treatment period.

Despite these limitations, this study provides new empirical support for the function of oral output modality to listening competence development which, to date, has been largely assumed but remained unclear with respect to specific linguistic skills. In addition to expanding the theoretical scope of output as a crucial construct in L2 acquisition, the findings of this research also contribute to promoting more modalities-integration perspectives of SLA (Tavil, 2010; Siegel, 2014). The different facilitation effects evidenced in the listening tasks shed light on L2 pedagogy, insightful for researchers and practitioners to flexibly employ oral output according to certain listening competence to be cultured. Also, the novel format takes a positive effect on promoting L2er’s class engagement and self-agency. Future research may examine the format’s effect on L2 outcomes in different L1 contexts and age groups.

## Data Availability Statement

The original contributions presented in this study are included in the article/Supplementary Material, further inquiries can be directed to the corresponding author.

## Ethics Statement

The studies involving human participants were reviewed and approved by the Shanxi Agricultural University. The patients/participants provided their written informed consent to participate in this study.

## Author Contributions

Both authors listed have made a substantial, direct, and intellectual contribution to the work, and approved it for publication.

## Conflict of Interest

The authors declare that the research was conducted in the absence of any commercial or financial relationships that could be construed as a potential conflict of interest.

## Publisher’s Note

All claims expressed in this article are solely those of the authors and do not necessarily represent those of their affiliated organizations, or those of the publisher, the editors and the reviewers. Any product that may be evaluated in this article, or claim that may be made by its manufacturer, is not guaranteed or endorsed by the publisher.

## Footnotes

1 Language-related episodes, or LREs means “any part of the dialog where the students talk about the language they are producing, question their language use, or correct themselves or others” (Swain and Lapkin, 1998, p. 326).

2 E.g., https://www.youtube.com/watch?v=N3glLccSYXc

3
https://speechling.com/zh/dictation/english

4
https://www.examenglish.com/PTE/pte_general_level3_listening_part1.htm

5
https://www.youtube.com/watch?v=EvhZPWJSub4

## References

[B1] Adrada-Rafael S. (2017). Processing the spanish imperfect subjunctive: depth of processing under different instructional conditions. *Appl. Psychol.* 38 477–508.

[B2] Anderson J. R. (1995). *Learning and memory: An integrated approach.* Hoboken: John Wiley & Sons.

[B3] Bernhardt S. (1997). Self-efficacy and second language learning. *NCLRC Lang. Res.* 1 1–13.

[B4] Bonyadi A. Nikou F. R. Shahbaz S. (2012). The relationship between EFL learners’ self-efficacy beliefs and their language learning strategy use. *Eng. Lang. Teach.* 5 113–121. 10.3389/fpsyg.2022.867560 35496223 PMC9040706

[B5] Brown H. D. Lee H. (2015). *Teaching by principles: An interactive approach to language pedagogy* (4th ed.). Boston: Pearson Education, Inc.

[B6] Brown S. Brown S. R. (2011). *Listening myths: Applying second language research to classroom teaching.* Ann Arbor: University of Michigan Press.

[B7] Bruderer A. G. Danielson D. K. Kandhadai P. Werker J. F. (2015). Sensorimotor influences on speech perception in infancy. *Proc. Nat. Acad. Sci.* 112 13531–13536. 10.1073/pnas.1508631112 26460030 PMC4640749

[B8] Buck G. (2001). *Assessing listening.* Cambridge: Cambridge University Press.

[B9] Callan D. E. Jones J. A. Callan A. M. Akahane-Yamada R. (2004). Phonetic perceptual identification by native-and second-language speakers differentially activates brain regions involved with acoustic phonetic processing and those involved with articulatory–auditory/orosensory internal models. *NeuroImage* 22 1182–1194. 10.1016/j.neuroimage.2004.03.006 15219590

[B10] Chun D. M. Hardison D. M. Pennington M. C. (2008). “Technologies for prosody in context: Past and future of L2 research and practice,” in *Phonology and second language acquisition*, eds Jette G. Hansen Ed Mary L. Z. (Cambridge: Cambridge University Press), 323–346.

[B11] DeKeyser R. M. (1998). “Beyond focus on form: Cognitive perspectives on learning and practicing second language grammar,” in *Focus on form in classroom second language acquisition*, eds Doughty C. Williams J. (Cambridge: Cambridge University Press).

[B12] DeKeyser R. M. (2001). “Automaticity and automatization,” in *Cognition and second language instruction*, ed. Peter Robinson (Cambridge: Cambridge University Press), 125–151.

[B13] DeKeyser R. M. (2007). *Introduction: Situating the concept of practice.* Cambridge: Cambridge University Press.

[B14] Essay. (2018). *Chinese communication style.* Nottingham: UKEssays.

[B15] Gandour J. Tong Y. Talavage T. Wong D. Dzemidzic M. Xu Y. (2007). Neural basis of first and second language processing of sentence−level linguistic prosody. *Human Brain Mapp.* 28 94–108. 10.1002/hbm.20255 16718651 PMC6871414

[B16] Garrison D. R. (2016). *E-learning in the 21st century: A community of inquiry framework for research and practice.* Abingdon: Routledge.

[B17] Gass S. M. (2013). *Input, interaction, and the second language learner.* Abingdon: Routledge.

[B18] Greenslade T. Bouden L. Sanz C. (1999). Attending to form and content in processing L2 reading texts. *Spanish Appl. Linguist.* 3 65–90.

[B19] Hakuta K. McLaughlin B. (1996). *Seven tensions that define research on bilingualism and second language acquisition. The Handbook of Educational Psychology.* Washington, DC: American Psychological Association.

[B20] Haslam M. (2018). *Teaching the sound system of English.* New York, NY: John Wiley & Sons, Inc.

[B21] Hinkel E. (2010). “Integrating the four skills: Current and historical perspectives,” in *The oxford handbook of applied linguistics*, eds Robert B. Kaplan (Oxford: Oxford University Press), 110–126.

[B22] Hubbard P. (2021). *An invitation to CALL: Foundations of computer-assisted language learning.* Taoyuan: APACALL.

[B23] Hulstijn J. H. Laufer B. (2001). Some empirical evidence for the involvement load hypothesis in vocabulary acquisition. *Lang. Learn.* 51 539–558.

[B24] Izumi S. (2002). Output, input enhancement, and the noticing hypothesis: An experimental study on ESL relativization. *Stud. Second Lang. Acquis.* 24 541–577.

[B25] Izumi Y. Izumi S. (2004). Investigating the effects of oral output on the learning of relative clauses in English: issues in the psycholinguistic requirements for effective output tasks. *Can. Modern Lang. Rev.* 60 587–609.

[B26] Kassem H. M. (2015). The relationship between listening strategies used by Egyptian EFL college sophomores and their listening comprehension and self-efficacy. *Eng. Lang. Teach.* 8 153–169.

[B27] Kato S. Tanaka K. (2015). Reading aloud performance and listening ability in an L2: the case of college-level Japanese EFL users. *Open J. Modern Ling.* 5:187.

[B28] Krashen S. D. (1985). *The Input Hypothesis.* Harlow: Longman.

[B29] Krashen S. D. (1992). *The Input Hypothesis: An Update. Linguistics and Language Pedagogy: The State of the Art.* Harlow: Longman. 409–431.

[B30] Lenneberg E. H. (1967). *The biological foundations of language.* New York, NY: Wiley.

[B31] Leow R. P. (1997). Attention, awareness, and foreign language behavior. *Lang. Learn.* 47 467–505.

[B32] Leow R. P. (2015). *Explicit learning in the L2 classroom.* Abingdon: Routledge.

[B33] Leow R. P. Mercer J. D. (2015). Depth of processing in L2 learning: theory, research, and pedagogy. *J. Span. Lang. Teach.* 2 69–82. 10.1080/23247797.2015.1026644

[B34] Levelt W. J. M. (1999). “Producing spoken language: A blueprint of the speaker,” in *The neurocognition of language*, eds Brown C. M. Hagoort P. (Oxford: Oxford University Press), 94–122.

[B35] Linebaugh G. Roche T. B. (2015). Evidence that L2 production training can enhance perception. *J. Acad. Lang. Learn.* 9 A1–A17.

[B36] Long M. (1996). *The role of the linguistic environment in second language acquisition. Handbook of Second Language Acquisition.* New York, NY: Academic Press.

[B37] Long M. H. (2006). *Problems in SLA. second language acquisition research series.* New York, NY: Lawrence Erlbaum Associates.

[B38] Lynch T. (2009). *Teaching second language listening* (1st ed.). Oxford: Oxford Univ. Press.

[B39] McLaughlin B. (2013). *Second language acquisition in childhood: Volume 2: School-age children.* London: Psychology Press.

[B40] Mede E. Dikilitaş K. (2015). Teaching and learning sociolinguistic competence: teachers’ critical perceptions. *Part. Edu. Res.* 2 14–31. 10.17275/per.15.29.2.3

[B41] Möttönen R. Watkins K. E. (2009). Motor representations of articulators contribute to categorical perception of speech sounds. *J. Neurosci.* 29 9819–9825. 10.1523/JNEUROSCI.6018-08.2009 19657034 PMC6666584

[B42] Nguyen H. Abbott M. (2016). Promoting process-oriented listening instruction in the ESL classroom. *TESL Can. J.* 34 72–86.

[B43] Oxford R. L. (1990). *Language learning strategies.* New York, NY: Newbury House.

[B44] Oxford R. L. (2003). *Language learning styles and strategies: An overview.* Oxford: GALA.

[B45] Park E. S. (2013). Learner-generated noticing behavior by novice learners: tracing the effects of learners’ L1 on their emerging L2. *Appl. Ling.* 34 74–98.

[B46] Pica T. (1994). Research on negotiation: what does it reveal about second−language learning conditions, processes, and outcomes? *Lang. Learn.* 44 493–527.

[B47] Richards J. C. (2004). *Tactics for listening-developing: Student book.* Oxford: Oxford University.

[B48] Rosa E. M. Leow R. P. (2004). Awareness, different learning conditions, and second language development. *Appl. Psycholinguist.* 25 269–292.

[B49] Russell V. (2014). A closer look at the output hypothesis: the effect of pushed output on noticing and inductive learning of the Spanish future tense. *Foreign Lang. Ann.* 47 25–47. 10.1111/flan.12077

[B50] Sams M. Möttönen R. Sihvonen T. (2005). Seeing and hearing others and oneself talk. *Cogn. Brain Res.* 23 429–435. 10.1016/j.cogbrainres.2004.11.006 15820649

[B51] Schmidt R. (1992). Awareness and second language acquisition. *Ann. Rev. Appl. Ling.* 13 206–226. 10.1017/S0267190500002476

[B52] Schmidt R. (1994). Implicit learning and the cognitive unconscious: Of artificial grammars and SLA. *Impl. Expl. Learn. Lang.* 22 165–209.

[B53] Schmidt R. (2001). “Attention,” in *Cognition and second language instruction*, ed. Robinson P. (Cambridge: Cambridge University Press), 3–32. 10.1017/CBO9781139524780.003

[B54] Schmidt R. W. (1990). The role of consciousness in second language learning. *Appl. Linguist.* 11 129–158.

[B55] Schmidt R. Frota S. N. (1986). “Developing basic conversational ability in a second language: A case study of an adult learner of Portuguese,” in *Talking to learn: Conversation in second language acquisition*, ed. Day R. (New York, NY: Newbury House).

[B56] Siegel J. (2014). Exploring L2 listening instruction: Examinations of practice. *ELT J.* 68 22–30.

[B57] Siegel J. Siegel A. (2015). Getting to the bottom of L2 listening instruction: making a case for bottom-up activities. *Stud. Sec. Lang. Learn. Teach.* 5 637–662. 10.14746/ssllt.2015.5.4.6

[B58] Skehan P. (1998). *A cognitive approach to language learning.* Oxford: Oxford University Press.

[B59] Skehan P. Foster P. (2001). *Cognition and tasks. Cognition and Second Language Instruction.* Cambridge: Cambridge University Press. 183–205.

[B60] Son M. Lee J. Godfroid A. (2021). Attention to form and meaning revisited: Insights from eye tracking. *Stud. Sec. Lang. Acquis.* 2021 1–30. 10.1017/S0272263121000565

[B61] Swain M. (1985). Communicative competence: Some roles of comprehensible input and comprehensible output in its development. *Input Sec. Lang. Acquis.* 15 165–179.

[B62] Swain M. (1995). *Three functions of output in second language learning.* Oxford: Oxford University Press.

[B63] Swain M. (2000). *The output hypothesis and beyond: Mediating acquisition through collaborative dialogue.* Abingdon: Routledge.

[B64] Swain M. (2005). *The output hypothesis: Theory and research* (1st ed.). Abingdon: Routledge, 10.4324/9781410612700

[B65] Swain M. Lapkin S. (1998). Interaction and second language learning: Two adolescent French immersion students working together. *Mod. Lang. J.* 82 320–337.

[B66] Sydorenko T. Hellermann J. Thorne S. L. Howe V. (2019). Mobile augmented reality and language−related episodes. *TESOL Quart.* 53 712–740.

[B67] Tavil Z. M. (2010). Integrating listening and speaking skills to facilitate English language learners’ communicative competence. *Proc. Soc. Behav. Sci.* 9 765–770. 10.1016/j.sbspro.2010.12.231

[B68] Twyford C. W. (1987). *Age-related factors in second language acquisition.* Houston: Bilingual Education.

[B69] Vandergrift L. (2007). Recent developments in second and foreign language listening comprehension research. *Lang. Teach.* 40 191–210. 10.1017/S0261444807004338

[B70] Vandergrift L. Tafaghodtari M. H. (2010). Teaching L2 learners how to listen does make a difference: an empirical study. *Lang. Learn.* 60 470–497. 10.1111/j.1467-9922.2009

[B71] Vandergrift L. Goh C. C. M. Mareschal C. J. Tafaghodtari M. H. (2006). The metacognitive awareness listening questionnaire: development and validation. *Lang. Learn.* 56 431–462. 10.1111/j.1467-9922.2006.00373.x

[B72] VanPatten B. (1990). Attending to form and content in the input: An experiment in consciousness. *Stud. Sec. Lang. Acquis.* 12 287–301.

[B73] VanPatten B. (1993). Grammar teaching for the acquisition-rich classroom. *Foreign Lang. Ann.* 26 435–450. 10.1111/j.1944-9720

[B74] VanPatten B. (1996). *Input processing and second language acquisition: On the relationship between form and meaning.* New York, NY: McGraw-Hill.

[B75] VanPatten B. Williams J. (2015). “Input processing in adult SLA,” in *Theories in second language acquisition*, 2nd Edn, eds VanPatten B. Williams J. (Abingdon: Routledge), 125–146. 10.4324/9780203628942-12

[B76] Wilson J. (2018). *Macro listening skills.* New York, NY: John Wiley & Sons, Inc, 10.1002/9781118784235.eelt0571

[B77] Wong W. (2001). Modality and attention to meaning and form in the input. *Stud. Sec. Lang. Acquis.* 23 345–368.

[B78] Wu Y. (2019). Review of Chinese English learners’ prosodic acquisition. *Eng. Lang. Teach.* 12 89–94.

[B79] Yang J. C. Chang P. (2014). Captions and reduced forms instruction: the impact on EFL students’ listening comprehension. *ReCALL* 26 44–61. 10.1017/S0958344013000219

[B80] Yu J. Gibbon D. (2015). “How natural is Chinese L2 English prosody?” *Paper presented at the ICPhS.* Melbourne.

[B81] Zalbidea J. (2021). On the scope of output in SLA: task modality, salience, L2 grammar noticing, and development. *Stud. Sec. Lang. Acquis.* 43 50–82. 10.1017/S0272263120000261

