# Supplementary material for: Teaching EFLLs Listening Subskills With a Speaking-Listening Model in a Computer-Mediated Communication Setting
Source: Front Psychol. 2022 Jul 1;13:836013. doi: 10.3389/fpsyg.2022.836013 (PMC9284103; doi:10.3389/fpsyg.2022.836013)
Supplement: Supplementary file 1 [file Data_Sheet_1.DOCX]

# Listening comprehension Test

Part1: *You will have 10 seconds to read each question and the corresponding options. Then listen to the recording. After the recording you will have 10 seconds to choose the correct option. There are 10 questions in this section.*

1. Why does the woman think the kangaroo is a symbol of Australia?
2. She thinks it’s because there are a lot of them.
3. She says it’s because they are native animals.
4. She is not really sure about the true reason.
5. What are the two people discussing?
6. a stage play featuring animals
7. good and bad points of zoos
8. nature programmes on TV
9. Which animal is the man talking about?
10. cats
11. snakes
12. parrots
13. How much should the woman pay?
14. £2.60
15. £2.85
16. £2.50
17. What is the advertisement for?
18. a newspaper
19. a magazine
20. a TV programme
21. The woman thinks newspapers are …
22. rather boring.
23. full of information.
24. too expensive.
25. The man thinks the role of drummers in bands is …
26. questionable.
27. unimportant.
28. essential.
29. How does the man feel?
30. pleased
31. shocked
32. uninterested
33. The woman says the concert …
34. will be continuous.
35. is starting early.
36. may be a long one.
37. Where does the woman work?
38. a music shop
39. a box office
40. a restaurant

Part 2 *Listen to a conversation between a professor and a student.*

1. Why does the student go to see the professor?
2. To ask for help with a computer problem
3. To ask for advice on a project
4. To request more time to complete a paper
5. To explain her research proposal
6. Which of the following is not true?
7. The professor only accepts late papers under special circumstances.
8. The student still has time to completer the assignment.
9. The student lost all of her work when she lost her computer.
10. The student believes she won’t have time to completer the assignment.
11. What does the professor offer to do?
12. Call local taxi companies.
13. Lend her the use of a computer.
14. Extend the project deadline.
15. Make time to meet with the student

Part 3 *Listen to the conversation. Write the missing words.(twice)*

A: So what (14.)__________________________ this weekend?

B: My friends and I went to Los Angeles for the weekend.

A: Oh, really? Did (15.)______________________famous?

B: Yeah! We saw Leonardo DiCaprio in a cafe and Jackie Chan walking down the street!

A: No way! Did you get their autographs?

B: No. We were too shy to ask. What (16.)_____________________if you say someone famous? Would you ask for an autograph?

A: Maybe. I was at a restaurant once, and I took a picture(17.)________________________celebrity.

## Part 4

*Complete the sentences below. Write no more than two words for each answer.*

1. According to UNESCO, the Forbidden city has the greatest number of _____________in the world.
2. One of the most significant colors in the architecture of the Forbidden City is _______________.
3. Black is used for the ____________of one building because it represents water.
4. The number and layout of buildings in the Forbidden City are intended to represent the _____________ and _______________.

注意： 务必将答案写在答题纸上！

**Answer sheet**

**Part 1**

1. A B C
2. A B C
3. A B C
4. A B C
5. A B C
6. A B C
7. A B C
8. A B C
9. A B C
10. A B C

**Part 2**

1. A B C D
2. A B C D
3. A B C D

**Part 3**

1. _______________________________
2. _______________________________
3. _______________________________
4. ________________________________

**Part 4**

1. _______________________________
2. _______________________________
3. _______________________________
4. _______________and _________________
