# Supplementary material for: Teaching EFLLs Listening Subskills With a Speaking-Listening Model in a Computer-Mediated Communication Setting
Source: Front Psychol. 2022 Jul 1;13:836013. doi: 10.3389/fpsyg.2022.836013 (PMC9284103; doi:10.3389/fpsyg.2022.836013)
Supplement: Supplementary file 2 [file Data_Sheet_2.PDF]

English Learner Questionnaire

The purpose of this survey is to better understand English learners' thoughts and beliefs about our listening and speaking course. The questionnaire is not a test so there are no "right" or "wrong" answers. We are interested in your personal opinion. The results of this survey will be used only for research purpose. Please give your answers sincerely. Thank you very much for your help.

热忱邀请你参与本次英语学习调研。你的想法和判断没有对错之分，不会影响到你的学习成绩，但将会影响到今后英语听说课的教学改革方向。感谢你的宝贵想法和建议！

Student Information

Name 姓名:____________ Age 年龄:______ Gender 性别: M 男 F 女

Are you majoring in English: （英语专业）yes / （非英语专业）no

Year of studying English 英语学习年限: ___________________

English proficiency 英语水平: 高 high / 中 intermediate / 低 low（感觉这部分没必要，因为全是非英语专业的）

Part I Please tell us how much you agree or disagree with the following statements by simply tickle the number that best describe you when listening. **请根据实际情况选择相应的数字来表达同意或不同意的程度。**

Strongly disagree	Disagree	Slightly disagree	Slightly Agree	Agree	Strongly agree
**1** **强烈不同意**	**2** **不同意**	**3** **基本不同意**	**4** **基本同意**	**5** **同意**	**6** **强烈同意**

(*Example*) If you strongly agree with the following statement, write this:√

比如： 要是强烈同意以下表达，就在相应的字母代表处划"√"

I like eating ice cream very much. 我超喜欢吃冰激凌。 **1 2 3 4 5 6√**

1. I listen to what is said without paying much attention to every new word.<br>我专注于所说内容，不会纠结每个生词的意思。	**1 2 3 4 5 6**
2. I know the way to stress one syllable in a word is to make it louder, higher and longer.<br>我知道重音音节的发声规律，即声音较大些，音调较高些，用时较长些。	**1 2 3 4 5 6**
3. I use my experience and knowledge to help me understand.<br>我会结合自己的知识和经验来理解和判断所听材料基本意思。	**1 2 3 4 5 6**

4. When there's something I don't understand, I pursue trying to compensate for it.<br>我不会因为某一地方听不懂而卡住，相反，我会继续听，到后面就懂了。	**1 2 3 4 5 6**
5. I skip over words that I do not understand so that I don't miss what is said next.<br>我会跳过听不懂的单词，这样我不会错过接下来要听的信息。	**1 2 3 4 5 6**
6. I like to share my newly discovered valuable English video materials with my classmates.<br>我喜欢和同学分享我最新发现的好的英文视听材料。	**1 2 3 4 5 6**
7. I practice sounds in the target language that are very different from sounds in my own language to become familiar with them.<br>我会经常练习英语的语音语调，好让自己对英语发音规律更为熟悉。	**1 2 3 4 5 6**
8. I use the main idea of the text to help me guess the meaning of the words that I don't understand.<br>我能够结合文章主题来猜测某些生词的意思。	**1 2 3 4 5 6**
9. I try to relax whenever I feel tense as I listen.<br>做听力有时会紧张，我会尝试着放松心情。	**1 2 3 4 5 6**
10. I use pronunciation aspects like stress and intonation to enhance my understanding.<br>我会结合发音规则如重音、语调知识来协助我准确理解所听内容。	**1 2 3 4 5 6**
11. After listening, I evaluate how much I could understand.<br>每听完一则听力，我习惯于评估一下自己听懂了多少。	**1 2 3 4 5 6**
12. I use the speakers' tone and intonation to better understand what I listen to.<br>我能从说话人的语气、语调来判断他们要表达的意思。	**1 2 3 4 5 6**
13. I translate in my head as I listen.<br>听英语时，我会在心里将其翻译为汉语，以助于理解。	**1 2 3 4 5 6**
14. When I compare myself to other students in my class, I'm a good listener.<br>与班上其他同学相比，我的听力还是不错的。	**1 2 3 4 5 6**
15. I know retaining collocations can help me understand native speakers.<br>我所记住的那些固定搭配可以协助我更好地理解原声英语。	**1 2 3 4 5 6**
16. I can handle more challenging listening materials than I could before.<br>相比之前，现在我所听的英语材料更有挑战，但我可以听懂。	**1 2 3 4 5 6**
17. I watch movies in the target language.<br>通过经常观看原声英语电影来提高英语听力理解能力。	**1 2 3 4 5 6**
18. When I listen, I can answer more questions than other students.<br>听力课上，比起其他同学，我能回答对更多的问题。	**1 2 3 4 5 6**
19. I understand what I listen to better than I could before.<br>我发现我现在比以前能听懂的东西多了。	**1 2 3 4 5 6**
20. When I find listening difficult, I usually give up.<br>一旦遇到听不懂的情况，我就干脆不听了。	**1 2 3 4 5 6**
21. I know what strategies to use when I listen to English.<br>我能选择对应的策略来协助我听懂英语。	**1 2 3 4 5 6**
22. I identify my problems in listening and work on solving them.<br>我清楚哪些因素影响和制约我听力水平的提高，并在努力设法攻克。	**1 2 3 4 5 6**
23. I can understand what I listen to even if I don't know several vocabulary items.<br>有个别生词听不懂不要紧，我还是可以把握所听材料的意思的。	**1 2 3 4 5 6**

24. I like to listen to the familiar material with a faster speed.<br>针对那些熟悉的内容，我愿意把听力材料语速调快来训练我的听力。	**1 2 3 4 5 6**
25. I talk to my teacher and classmates about how to be a better listener of English.<br>我会和老师、同学探讨提高听力的方法。	**1 2 3 4 5 6**
26. I concentrate my attention on the key words not the fillers or pauses.<br>停顿、语气词、助词之类我会主动过滤掉，集中注意力听关键词的意思。	**1 2 3 4 5 6**
27. I know the key words in a sentence are uttered longer, higher and louder.<br>句子中的关键词通常会被讲话者以提高音量、音调，放慢语速来加以强调。	**1 2 3 4 5 6**
28. I know the flow of speech is made by contracting words together into idea units.<br>流利的口语通过连读、略读等办法来达到意群内部单词黏连在一起的目的。	**1 2 3 4 5 6**
29. I take notes when listening. 我习惯边听边记笔记。	**1 2 3 4 5 6**
30. I can mimicry the rhythm of spoken sound.<br>模仿语料时，我能够感受到原声英语的节奏、鼓点。	**1 2 3 4 5 6**
31. I realize that words can be linked together for the sake of easy vocalizing.<br>便于发声，单词和单词往往要黏在一起而不是单独发音。	**1 2 3 4 5 6**
32. I try to memorize the ideas when listen to a long speech.<br>听较长篇幅听力时，我会尝试记住部分观点，方便理解通篇意思。	**1 2 3 4 5 6**
33. I can tell the speaker’s attitude by discriminating his/her rising or lowering pitch.<br>通过判断说话人语调能有效推断出说话人的态度。	**1 2 3 4 5 6**
34. I can recognize cohesive devices in spoken discourse.<br>我能够敏锐捕获听力语篇中的连接词。	**1 2 3 4 5 6**
35. I chunk the spoken sound into smaller units according to melodic features.<br>根据说话人声音节奏上的变化，我可以将语段划分成小片段来加以理解。	**1 2 3 4 5 6**
36. I can infer situations, participants, goals using real-world knowledge.<br>根据自己的知识框架，我可以推断出所听内容的情景、人物关系、交际目的。	**1 2 3 4 5 6**
37. I distinguish major parts and minor parts when I listen.<br>听英语时，我很清楚哪些地方要重点听，哪些地方可以忽略掉。	**1 2 3 4 5 6**
38. I identify the communicative functions according to situations, participants and goals.<br>根据所涉及情景、人物及目的，我能听出句子的交际功能是感谢还是道歉等。	**1 2 3 4 5 6**

Adapted from Kassem, H. (2015), Brown (2014).
